# Visual detection and differentiation of Classic Swine Fever Virus strains using nucleic acid sequence-based amplification (NASBA) and G-quadruplex DNAzyme assay

**DOI:** 10.1038/srep44211

**Published:** 2017-03-13

**Authors:** Xiaolu Lu, Xueyao Shi, Gege Wu, Tiantian Wu, Rui Qin, Yi Wang

**Affiliations:** 1 School of Environmental Studies, China University of Geosciences (Wuhan), Wuhan 430074, P. R. China; 2 College of Life Sciences, South-central University for Nationalities, Wuhan 430074, P.R. China

## Abstract

The split G-quadruplex DNAzyme has emerged as a valuable tool for visual DNA detection. Here, we successfully integrated colorimetric split G-quadruplex DNAzyme assay with nucleic acid sequence-based amplification to generate a novel detection approach, allowing visual and rapid detection for the RNA of Shimen and HCLV strains of Classic Swine Fever Virus (CSFV). CSFV is a RNA virus that causes a highly contagious disease in domestic pigs and wild boar. With this method, we were able to detect as little as 10 copies/ml of CSF viral RNA within 3 h in serum samples taken from the field. No interference was encountered in the amplification and detection of Classic Swine Fever Virus in the presence of non-target RNA or DNA. Moreover, Shimen and HCLV strains of Classic Swine Fever Virus could be easily differentiated using the NASBA-DNAzyme system. These findings indicate the NASBA-DNAzyme system is a rapid and practical technique for detecting and discriminating CSFV strains and may be applied to the detection of other RNA viruses.

Sensitive method for nucleic acid detection is very crucial for clinical diagnosis and virus identification. However, many of these strategies are either time-consuming or require sophisticated instruments that may not be available in laboratories with fewer resources. More sensitive, simple and rapid strategy for nucleic acid detection will always be in demand for the laboratory and point-of-care diagnostic applications. Recently, visual nucleic acid detection has attracted a lot of attention because of its satisfactory sensitivity, simplicity and portability^1^,^2^,^3^,^4^. G-quadruplex DNAzyme serves as a valuable tool for visual nucleic acid detection.

The G-quadruplex/hemin complex, acting as an activated horseradish peroxidase (HRP)-mimicking DNAzyme, can catalyze the oxidation of 2,2′-azino-bis (3-ethylbenzthiazoline-6-sulfonic acid) (ABTS^2−^) by H_2_O_2_ to produce a colorimetric signal that can be visualized by the naked eye^5^,^6^,^7^,^8^,^9^. The visualization strategy based on the DNAzyme has been used for direct detection of pathogen DNA, such as DNA biomarkers of human immunodeficiency virus (HIV)^4^,^10^ as well as DNA of some pathogenic bacteria^11^,^12^. More importantly, the split G-quadruplex DNAzyme strategy can distinguish single base differences and has been used for the differentiation of gene mutations in *Mycobacterium tuberculosis*^11^. Nucleic acid sequence-based amplification (NASBA) is a homogeneous, isothermal RNA amplification process that can be used for the continuous amplification of RNA in a single mixture at one temperature^13^. Its advantage over the traditional RT-PCR technique is its suitability for the direct amplification of RNA targets, obviating the need for thermal cycling equipment. NASBA has been successfully used for the detection of many pathogens, such as *Staphylococcus aureus*^14^, the Salmonella genus^15^, human immunodeficiency virus^16^, human papillomavirus^17^, etc. In this paper, a novel strategy was developed by combining NASBA technique with colorimetric split G-quadruplex DNAzyme system for specific and sensitive detection of a RNA virus, CSFV.

Classic swine fever virus (CSFV) is the etiologic agent of classic swine fever (CSF), which causes a highly contagious disease in domestic pigs and wild boar. Outbreaks of CSF usually lead to significant economic losses in many countries worldwide^18^. Different prevention and control strategies have been adopted to contain CSF in different parts of the world. In European countries, CSF has been successfully eradicated by a stamping-out policy since the 1990s^19^,^20^. For many countries outside Europe, such as China, vaccination is a major control strategy, with attenuated vaccines widely used because they induce excellent immune responses^21^. Most attenuated vaccines are based on the lapinized Chinese strain of hog cholera virus (HCLV), which are being safely and efficiently employed as prophylactics worldwide^22^. However, current vaccines on the market are unable to distinguish between the wild-type and vaccine strains of CSFV in vaccinated swine herds^22^.

Due to the big loss CSF may cause, many CSFV detection methods has been set up in pig herds. The two most-used assays are the virus neutralization test and ELISA. Although the virus neutralization test has been regarded as the “gold standard”, it is work-intensive and time-consuming, and thus not suitable for detection of large numbers of samples^23^. Besides, few methods can distinguish between field and vaccine strains of CSFV^24^. In the past, CSF has also been characterized by easily observable and typical symptoms such as fever and bleeding. Recently, however, these symptoms have been less obvious or absent in CSF cases. Therefore, it is necessary to develop a reliable and rapid method that can be used to screen pigs and can differentiate between field and vaccine strains of CSFV.

In this study, we utilized the colorimetric split G-quadruplex DNAzyme system combined with NASBA technique to generate a novel CSFV detection approach. This approach adopted a split probe targeting system, designed with G-rich sequences, which reassembles in the presence of target RNA amplified by NASBA, and producing G-quadruplexes with a catalytic activity. With this approach, we could detect Shimen strain (the most widespread and highly virulent wide type CSFV strain in China) in serum samples and distinguish them from the HCLV strain (the most used vaccine strain in China) within 3 hours through a simple colorimetric change.

## Results

### Design and construction of assay

**Figure 1:**
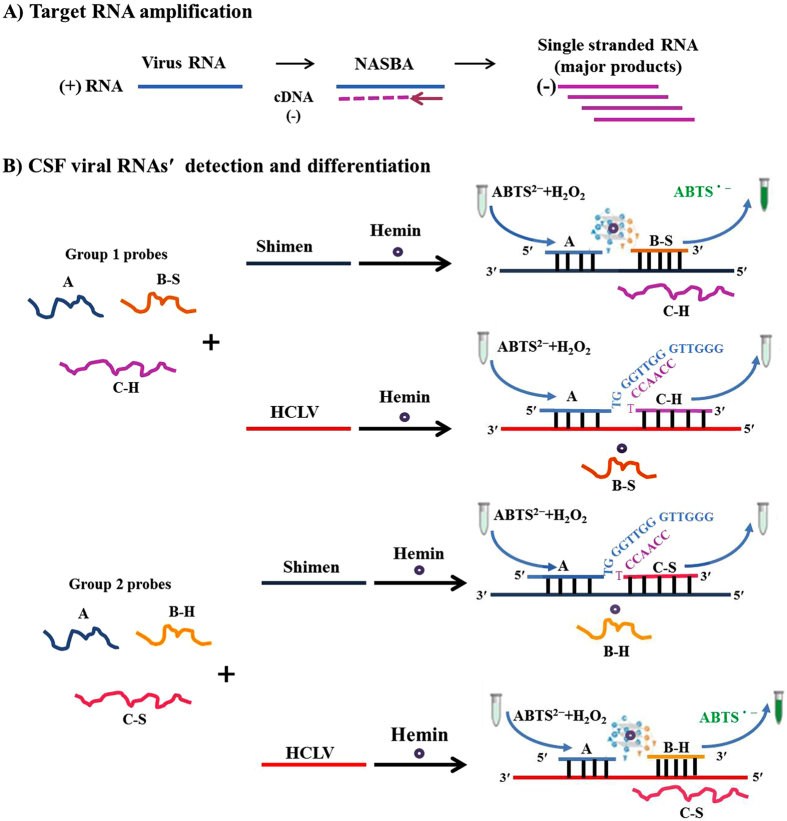
Principle of the NASBA-DNAzyme system for detection and differentiation of CSF viral RNAs.

DNAzyme system is a powerful technique in detecting DNA. In the present study, we tried to utilize this technology for fast and visual detection of RNA virus. The theory of the split peroxidase DNAzyme system had been introduced before^5^. In brief, when target DNA appears in the solution, probe A and B can be pulled together through hybridization with the adjacent DNA sequence in the target DNA, and a G-quadruplex structure will be formed by the overhanging sequences of probes A and B. When bound with hemin, the DNA G-quadruplex converts into a DNAzyme that possesses peroxidase-like activities and can catalyze H_2_O_2_ -mediated oxidization of ABTS^2−^, which results in the formation of the oxidation product ABTS and a discernible green color. In our study, viral RNA (CSFV RNA) was first extracted from the cell or serum samples. Then single-stranded target RNA was amplified by NASBA (Fig. 1A). To reduce the interference of the chromogenic reaction, a purification step was added following NASBA. Finally, probes were added to the system and hybridized with the purified target RNA, and thus formed the G-quadruplex structure to initiate the color reaction (Fig. 1B).

In order to differentiate wild-type strain from vaccine strain of CSFV, probes were carefully designed according to the genome sequence differences. Sequence alignment of Shimen and HCLV genome showed a distinctive difference in one conserved region in the coding region of the glycoprotein E2, which converted from “5′ CGUGACA 3′” (3430–3436nt) in Shimen strain to “5′ UGCGACU 3′” (3431–3437nt) in HCLV. Therefore, the 39 nt sequence of the CSF viral genome containing this sequence difference was used as the “detection sequences” to design our detection probes. We designed two groups of probes to detect the RNA of Shimen strain or the RNA of HCLV, each group contains three probes (Group 1 for Shimen: probes A, B-S, C-H; Group 2 for HCLV: probes A, B-H, C-S). The 5′ end of probe A has the same sequence as the 5′ end of the “detection sequences” both in Shimen´s and HCLV´s genome, and could hybridize with the 3′ end of the minus strand of the detection target DNA/RNA. The 3′ end of probe B-S or B-H could hybridize with 5′ end of the minus strand of detection target DNA/RNA of Shimen strain or HCLV. The 3′ end of probe A and the 5′ end of probe B-S or B-H could form a G-quadruplex structure when probe A and B were pulled together through hybridize with the adjacent sequence in target viral DNA/RNA. Probe C-H and C-S were designed as the competitive probes which are complementary with the minus strand of the detection target DNA/RNA of HCLV or Shimen, these two probes would compete with probes B-S and B-H in binding to the target DNA/RNA and thus may reduce false positive results (Fig. 1B). When using group 1 probes for Shimen virus detection, if the target DNA/RNA comes from Shimen strain, then probes A and B-S would play a significant role in bonding with the target nucleotide and would form an activated DNAzyme, producing a visible color change. If the target DNA/RNA comes from HCLV, probes A and C-H would bind to the target, but G-quadurplex could not be formed as well as the peroxidase activity. Therefore, the reaction remains colorless. If the target DNA/RNA is neither Shimen nor HCLV, no probes will hybrid with the target DNA/RNA, and the reaction will also be colorless (Fig. 1B). Similar situation will occur when using group 2 probes for detection, a color change will only be observed if HCLV DNA/RNA exist in the system (Fig. 1B).

### Detection of CSFV DNA

**Figure 2:**
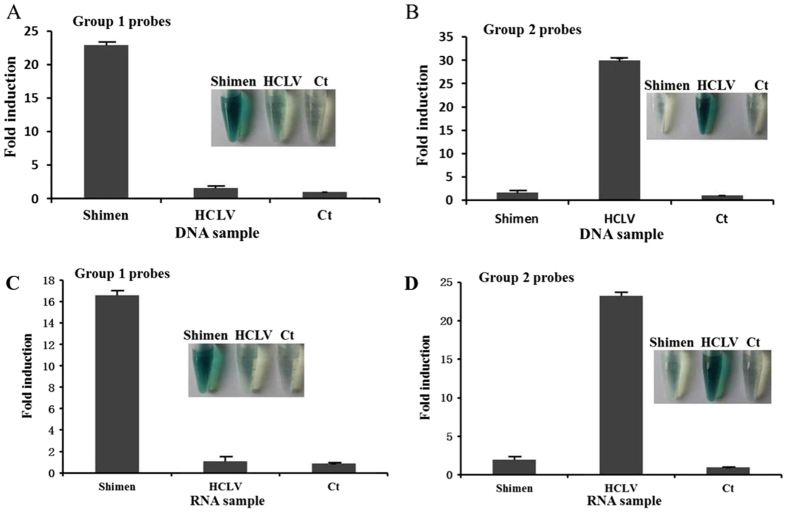
Detection of CSF viral DNA and RNA. CSF viral DNA and RNA samples were detected using group 1 probes for Shimen DNA (**A**) or Shimen RNA detection (**C**), and using group 2 probes for HCLV DNA (**B**) or HCLV RNA detection (**D**). Absorbance (OD) was measured at 414 nm and normalized to the value of negative control (Ct). Ct: negative control with no template added to the detection system. The inset is the corresponding photograph of the color change. The data represent the mean ± S.D. of three independent experiments.

To verify the effectiveness of the detection probes, we first tested the detection system using *in vitro* synthesized single-stranded DNA templates. The sequences of the DNA templates were listed in Table 1, and they had the same sequence as the minus strand of the target DNA/RNA. When using group 1 probes, a discernible green color change was observed with the sample of Shimen-DNA, while the sample of HCLV-DNA and the control showed very light green color (Fig. 2A). The optical density reading from the Shimen DNA detection was approximately 23 times stronger than that from the control, and was at least 10 times stronger than that from the HCLV DNA sample (Fig. 2A); Similarly, when using group 2 probes, the HCLV-DNA sample showed deep green color while Shimen-DNA sample or the control were almost colorless (Fig. 2B). The optical density reading from the HCLV DNA detection was almost 30 times stronger than the control when using group 2 probes, while negative result was obtained with Shimen DNA sample (Fig. 2B). These results indicated that this method could be used to detect and differentiate the target DNA of CSFV wild-type strain (Shimen) and vaccine strain (HCLV) clearly and efficiently.

### Parameter optimization

To obtain better detection sensitivity, some key parameters were optimized including the concentrations of hemin, ABTS^2−^, probes A&B, as well as different sequence compositions of A probes. The concentration of DNA templates used in the optimization experiments was 0.8 μM to obtain obvious color change. As shown in Fig. S1, the absorbance value increased as the concentration of hemin increased from 0.8 μM to 2 μM and reached the highest value at 1.6 μM, which indicated that 1.6 μM was the best concentration of hemin for detection. Similarly, the best concentration of probes for the detection was 400 nM (Fig. S2). The best amount of RNA target was 25 ng per sample when the concentrations of hemin and probes were 1.6 μM and 400 nM respectively (Fig. S3). Moreover, six A probes (A1-A6) with different sequence composition were designed in order to reduce the background signal. The sequence of different A probes are listed in Table 1. As shown in Figure S4, all six probes led to an obvious color change when using synthesized DNA template for detection. Within these probes, A6 showed the best sensitivity with 11.63 and 16.08 fold of increase of the absorbance value compared to the mock control (Fig. S4). In the following experiments, we used A6 for all the detections.

### Detection of CSFV RNA in virus-infected cells

Our visual system was further tested for its effectiveness in detecting CSF viral RNAs. Viral RNAs were extracted from virus-infected cell lysates and subjected to NASBA step to amplify the target region (241nt) into minus- and single-stranded RNA copies. Then probes, substrates, and reagents were add to the purified RNA templates and incubated for 30 sec before a subtle color change could be observed. When using group 1 probes for Shimen RNA detection, a discernible green color change was observed with the sample of Shimen-infected PK15 cells, while the samples of the mock-infected PK15 cells and HCLV-infected PK15 cells were almost colorless (Fig. 2C). Besides, the optical density reading from the sample of Shimen-infected PK15 cells was approximately 16 times stronger than that from the mock-infected or HCLV-infected PK15 cells samples (Fig. 2C). When using group 2 probes for HCLV RNA detection, the HCLV-infected cell sample showed deep green color while Shimen-infected or mock-infected cell samples gave out very light green color (Fig. 2D). The optical density reading from the HCLV-infected cell sample was more than 10 times stronger than that from the Shimen-infected cell sample (Fig. 2D). The results demonstrated that the detection system worked well with the RNA templates.

### Sensitivity

**Figure 3:**
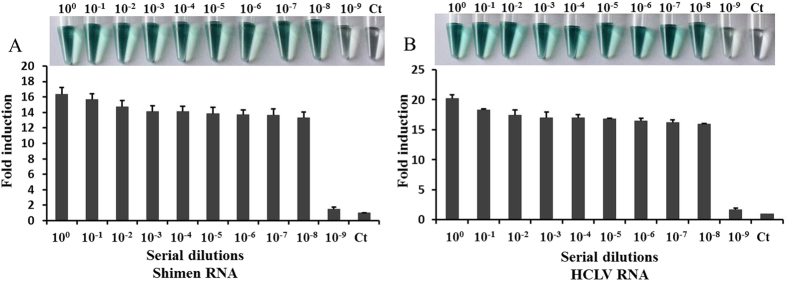
Detection sensitivity. 2 μg of total RNA extracted from Shimen- or HCLV-infected cells were serial 10-fold diluted, and subjected to detection for Shimen RNA using group 1 probes (**A**) or for HCLV RNA using group 2 probes (**B**). Absorbance (OD) was measured at 414 nm and normalized to the value of negative control (Ct). Ct: negative control with no template added to the detection system. The inset is the corresponding photograph of the color change. The data represent the mean ± S.D. of three independent experiments.

The detection limit of our detection system was evaluated by testing a virus RNA panel, which included 10-fold serial dilutions of Shimen-RNA and HCLV-RNA. The viral RNA concentrations of the cell samples were titrated by real-time RT-PCR before in our lab, with about 2.5 × 10^10^ copies/ml of Shimen-RNA and 2.5 × 10^10^ copies/ml of HCLV-RNA. As shown in Fig. 3, the detection signal generated by the NASBA-DNAzyme system was proportional to the dilution ratio with a detection limit of 10^8^ dilutions for Shimen strain (Fig. 3A) and 10^8^ dilutions for HCLV (Fig. 3B). No signal was obtained with negative controls. This minimum viral RNA concentration corresponds to as little as 10 copies/ml of Shimen RNA and 10 copies/ml of HCLV RNA if we consider that only 4 μl from each 100 μl viral dilution was added to the reaction mixture and amplified by NASBA. Compared with the detection limit of RT-PCR, which was 10^6^ dilutions for viral RNA with the same concentration (Fig. S5), these results indicated that the sensitivity of NASBA-DNAzyme system was 100 times better than RT-PCR.

### Specificity

**Figure 4:**
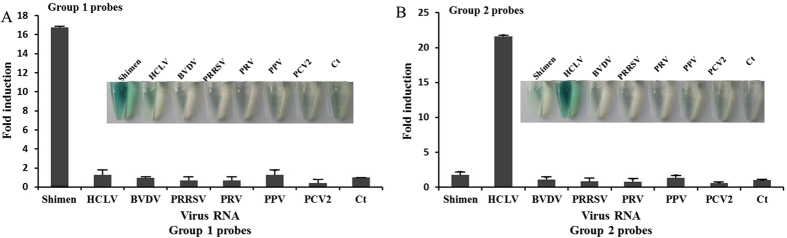
Detection specificity. 2 μg of total RNA extracted from different viruses infected cells were detected for Shimen RNA using group 1 probes (**A**), or tested for HCLV RNA using group 2 probes (**B**). Absorbance (OD) was measured at 414 nm and normalized to the value of negative control (Ct). Ct: negative control with no template added to the detection system. The inset is the corresponding photograph of the color change. The data represent the mean ± S.D. of three independent experiments.

Analytic specificity of our method was evaluated by testing a viral RNA panel, which was constituted with the RNA of several viruses commonly infecting pigs. The total RNA from the cells infected with CSFV Shimen and HCLV strains, 1 BVDV strain, 1 PRRSV strain, 1 PRV strain, 1 PPV strain, 1 PCVII strain were included in the panel. As shown in Fig. 4A,B, our method could detect the CSFV Shimen strain or the HCLV strain, while negative results were obtained with other virus strains. Three independent reproducibility tests were conducted and consistent results were obtained. Overall, the specificity and sensitivity of the NASBA-DNAzyme method showed that this method is a feasible detection tool for CSFV detection and differentiation.

### Detection of CSFV in serum samples

**Figure 5:**
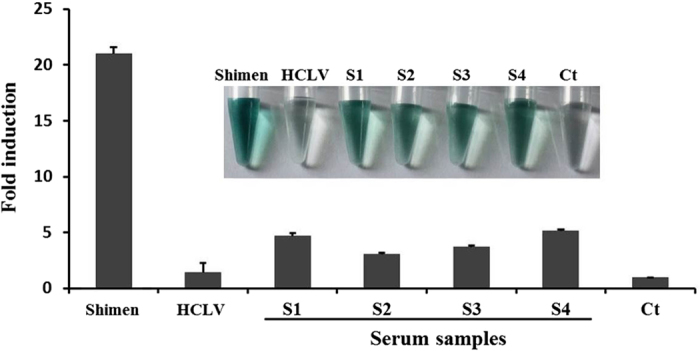
Detection of CSFV in serum samples CSF viral RNAs were detected from serum samples (S1–S4) using group 1 probes. Shimen and HCLV RNA were included to the detection. Absorbance (OD) was measured at 414 nm and normalized to the value of negative control (Ct). Ct: negative control with no template added to the detection system. The inset is the corresponding photograph of the color change. The data represent the mean ± S.D. of three independent experiments.

After the aforementioned optimization, the NASBA-DNAzyme method was applied to the detection of CSF viral RNA in serum samples. 4 serum samples from Shimen-infected pigs were tested. As shown in Fig. 5, NASBA-DNAzyme method could detect all four Shimen-infected blood samples with 3–5 fold increase of the OD values compared with the negative control, and a bright green color was observed in all these samples. Moreover, HCLV RNA sample gave only 1.46 fold increase of the OD value compared with the negative control and no obvious color change was observed in the assay. These results indicated that NASBA-DNAzyme method could detect Shimen strains as well as differentiate them from HCLV strain in serum samples.

## Discussion

We developed a novel method by integrating NASBA and split G-quadruplex DNAzyme analysis to detect and differentiate CSFV Shimen and HCLV strains. Traditional methods for CSFV detection relied on virus isolation or serological diagnosis. Recently, several molecular assays have been developed to detect CSFV and replace classical assays and are in wide use^25^,^26^,^27^. However, few of these methods could discriminate between wild-type CSFV strains and vaccine strains. Since the DNAzyme system could be used in detecting DNA of single base differences^11^, we adopted the DNAzyme system to our method and successfully detected viral RNA as well as differentiated between Shimen and HCLV strains of CSFV. Another important advantage of adopting the DNAzyme system in our method is the instant and visible color-change result without the need of signal readout from an apparatus.

DNAzyme system needs single-stranded nucleic acids to serve as the template. To obtain enough nucleic acid for detection, PCR procedure is often adopted before detection. However, the products of classical PCR are double-stranded and are not suitable for the DNAzyme system. Deng et al.^11^ used asymmetrical PCR to produce single-stranded templates in *Mycobacterium tuberculosis* detection, and nested PCR procedure were also added to the experiment due to the low efficiency of asymmetrical PCR, which increased the complexity of the detection method. In our study, single-stranded RNA templates were amplified through a one-step NASBA procedure, which largely simplified the detection steps. Besides, the RNA of CSFV was detected directly without reverse transcription and PCR step and no expensive reagents or apparatus were used compared with other molecular detection methods for RNA virus. The sensitivity of this method was enhanced because of NASBA and DNAzyme system, and was about 100 times more sensitive than the RT-PCR method.

As has been reported, DNAzyme system had been used for direct detection of DNA of many pathogens such as human immunodeficiency virus (HIV)^4^,^10^ and some pathogenic bacteria^11^,^12^. However, to the best of our knowledge, this system has never been applied for the detection of RNA virus. In the present study, we successfully used the DNAzyme system to detect RNA virus, which expand the application of this powerful technique in nucleic acid detection.

The ability of the method to detect and differentiate CSF viral RNA in serum samples suggested that it is applicable for field investigation of CSFV infection. The whole detection procedure could be completed within 3 hour without the use of complex instruments. Only a thermostatic water bath is needed. The results are visual, with the positive results indicated by a green color that is clearly observable by the naked eye. These advantages make this method an ideal option for field assays. Further works are still needed to simplify the purification procedure after NASBA and to apply the assay for other RNA virus detections.

In the present study, we aimed at establishing a new strategy for fast and sensitive detection of the most widespread wild-type CSFV strains in China, as well as discriminating them from the most used vaccine strain (HCLV). Therefore, we designed the detection probes according to a consensus sequence located in the conserved region of E2 gene, where a distinctive difference was found between 9 wild-type strains in China and HCLV, converting from “5′ CGUGACA 3′” in wild-type strains to “5′ UGCGACU 3′” in HCLV (Fig. S6A). The NASBA primers were also carefully designed to hybridize with exactly the same sequences in all 9 wild-type CSFV strains and HCLV. Our results showed successful detection and discrimination of CSFV Shimen and HCLV strains by these probes, which led us to believe that our detection system could be successfully applied for the detection and discrimination of any of these 9 wild-type strains in China from HCLV. However, when aligned the sequences of E2 gene between Shimen, HCLV and other widespread wild-type CSFV strains circulating outside China, a conserved “CGUGACU 3′” sequence was found in all wild-type strains outside China, which was different from the CSFV wild-type strains in China (Fig. S6B). This indicated that our probes might not suitable for the detection of the CSFV strains outside China. But our method should still work if we redesign a pair of pan-CSFV NASBA primers and specific probes according to the consensus sequence of other CSFV strains circulating globally.

In conclusion, this work has strongly confirmed the effectiveness of the NASBA-DNAzyme method in the detection and differentiation of CSFV RNA in cell and serum samples. This approach offers several advantages over other molecular techniques, including high sensitivity and specificity, rapid visual readout, ease-of-use, and inexpensiveness. In future, we will try to simplify the detection steps further and accommodate this method to the detection of other RNA viruses.

## Material and Methods

### Reagents

Hemin (Acrose), HEPES (Amresco), dimethyl sulfoxide (DMSO; Sinopharm Chemical Reagent Co., Ltd.), ABTS [2,2′-azino-bis(3-ethylbenzthiazoline-6-sulfonic acid) diammonium salt; Sigma-Aldrich], H_2_O_2_ (Sinopharm), Tris-HCl (Sinopharm), KCl (Sinopharm), NaCl (Sinopharm), T7 RNA Polymerase (Promega), AMV Reverse transcriptase (Promega), RNase H (Thermo Scientific), BAS (TaKaRa), M-MLV (Invitrogen). For the hemin solution, hemin was dissolved in DMSO to obtain a final concentration of 10 mM. For the ABTS solution, ABTS was dissolved in DMSO to obtain a final concentration of 250 mM.

### Equipment

The absorption spectrum of ABTS was measured at the wavelength of 414 nm using a SpectraMax i3 multiscan Spectrum (Molecular Devices, China). A Canon digital camera was used to take all the photographs in the experiment. A water bath (Jintan HH4, China) was used for the NASBA procedure.

### Viruses and cells

The cells infected with 2 CSFV strains (Shimen and HCLV), 1 BVDV strain (NADL), 1 PRRSV strain (Hubei), 1 PRV strain (HB), 1 PPV strain (HN99), 1 PCVII strain (HBZX) were provided by China Institute of Veterinary Drug Control.

### Preparation of viral RNA

RNAsimple Total RNA kit (TIANGEN, China) was used to extract total RNA from cell and serum samples, according to the manufacturer’s protocol. Ten-fold serial dilutions of RNA from virus isolates were prepared.

### NASBA procedure

The NASBA procedure used in this study has been previously described by Kievits^16^ with some modifications. The final volume of the reaction mixture was 20 μL. First, a 11 μL volume pre-reaction mixture was assembled so that the final concentration for 20 μL would be: 40 mM-Tris/HCl, pH 8.5, 12 mM-MgCl, 70 mM-KCl, 10 mm-DTT, 1 mM of each dNTP, 2 mM of each NTP, 15% (v/v) DMSO, 0.5 μM NASBA Primer 1 and 0.5 μM NASBA Primer 2. After addition of 4 μL of target RNA, the tubes were incubated at 65 °C for 5 min to uncoil the tertiary or secondary structures of the target RNA. Then the reaction mixtures were transferred to 41 °C for 3 min before 5 μL of an enzyme mix was added to the tubes, resulting in a final volume of 20 μ1, 0.1% BSA 2.4 μL, 16 U T7 RNA polymerase (Thermo scientific), 2.5 U AMV-RT (Promega), 0.1 U RNAase H (Thermo scientific). The mixtures were further incubated at 41 °C for 1.5 h for isothermal amplification of the RNA targets. Finally, the amplification products were purified with isopropanol and ethanol. In detail, 180 μL RNA free water, 300 μL isopropanol were added to 20 μL NASBA products and the mixture was incubated on ice for 15 min, followed by centrifuge at 13,000 g for 10 minutes at 4 °C. The supernatant was discarded and the pellet was rinsed with 1 mL 70% ethanol. The pellet was dissolved in 20 μL RNA free water.

### Probes and Primers

Probes were prepared at 4 °C as 20 μM stock solutions and then stored for several months at −20 °C. The probe mixtures for group detection were prepared as 5 μM stock solutions at 4 °C and stored for several months at −20 °C. The sequences of probes and primers are listed in Table. 1. All DNA oligonucleotides were synthesized by Shanghai Invitrogen Technology Co. Ltd. (Shanghai, China).

### Detection of viral DNA/RNA

NASBA products were added directly into the probe mixture containing 25 mM HEPES-NH_4_OH, 20 mM KCl, 200 mM NaCl, 400 nM probe A, 400 nM probe B, and 400 nM probe C for CSFV RNA detection. The final volume of mixtures were 97 μL adjusted by ultrapure water. The NASBA products were denatured at 65 °C for 3 min and then hybridized with probes at 40 °C for 3 min. Then 1.6 μL of 100 μM hemin was added to the system to ensure that the final concentration was 1.6 μM and kept at room temperature for 5 min. Then 2 μL of 50 mM ABTS and 0.15 μL of 1 M H_2_O_2_ were added to the system and incubated at room temperature for 2 min before the readout of the absorbance of the oxygenation product ABTS at 414 nm in a 100 μL reaction in a 96-well plate (Corning). Photograghs were immediately taken after colorimetric reaction. All reaction had been performed in triplicate to ensure reproducibility.

### Comparison with CSFV RT-PCR

The CSF viral RNAs from virus isolates (cell lysate) were also detected by RT-PCR (Reverse Transcription PCR) method. The reaction premix consisted of 5 μL of RNA free water, 5 μL of 10 μM forward primer, 5 μL of RNA template. The premix was incubated at 65 °C for 5 min and then on ice for 2 min. Then 2 μL of 10 mM dNTP, 100 U of M-MLV, 5 μL of M-MLV 5 × buffer and 5 μL of RNA free water were added to the premix, followed by incubation at 42 °C for 60 min and 75 °C for 10 min. 2 μL of the RT products were used as template for later PCR procedure. The PCR conditions were initial incubation at 94 °C for 2 min, followed by 30 cycles of denaturation at 94 °C for 30 sec, primer annealing at 60 °C for 30 s and extension at 72 °C for 30 sec. An addition step was conducted at 72 °C for 5 min.

## Additional Information

**How to cite this article:** Lu, X. *et al*. Visual detection and differentiation of Classic Swine Fever Virus strains using nucleic acid sequence-based amplification (NASBA) and G-quadruplex DNAzyme assay. *Sci. Rep.* **7**, 44211; doi: 10.1038/srep44211 (2017).

**Publisher's note:** Springer Nature remains neutral with regard to jurisdictional claims in published maps and institutional affiliations.

## Supplementary Material

Supplementary Information

## Figures and Tables

**Table 1:** Probes and Primers.

Probes and Primers	Sequence(5′-3′)	Description
T(−)DNA-S	GCGGTCTGTCACGTCCAGGTCAAACCAGTACTGATACTC	Target (−)DNA. The sequence differences are highlighted in red.
T(−)DNA-H	GCGGTCAGTCGCATCCAGGTCAAACCAGTACTGATACTC	
NASBA Primer 1	ACTATGAGCCCAGGGACAGCTACTT	Product size: 241 nt
NASBA Primer 2	GTCGACTAATACGACTCACTATAGGGTTCCCTATCAACACTACCTCACCCT	
RT-PCR Primer 1	CCATGCCCATAGTAGGACTAGCAAA	Product size: 105 bp
RT-PCR Primer 2	TCACGTCGAACTACTGACGACTGT	
Probe A	GAGTATCAGTACTGGTTT**TGGGTTGGGTTGGG**	Underline sequences are complementary to the target; **bold** sequences in probes A and B can form intermolecular G4 structure; *italic* sequences can hybridize with the **bold** sequence in probe A, inhibiting the intermolecular G4 structure formation between two probe A.
Probe B-S	**TGGGT**ACCTGGACGTGACAGACCGC	
Probe B-H	**TGGGT**ACCTGGATGCGACTGACCGC	
Probe C-S	*CCAACT*ACCTGGACGTGACAGACCGC	
Probe C-V	*CCAACT*ACCTGGATGCGACTGACCGC	
A1	GAGTATCAGTACTGGTTTTGGGTTGGGCAGGG	A probes for optimization.
A2	GAGTATCAGTACTGGTTTTGGGTTGGGACGGG	
A3	GAGTATCAGTACTGGTTTTGGGCAGGGTTGGG	
A4	GAGTATCAGCACTGGTTTTGGGTTGGGTTGGG	
A5	GAGTATCAGCACTGGTTTTGGGTTGGGCAGGG	
A6	GAGTATCAGTACTGGTTTTGGGTTGGGTTGGG	
