# Supplementary material for: Visual detection and differentiation of Classic Swine Fever Virus strains using nucleic acid sequence-based amplification (NASBA) and G-quadruplex DNAzyme assay
Source: Sci Rep. 2017 Mar 13;7:44211. doi: 10.1038/srep44211 (PMC5347003; doi:10.1038/srep44211)
Supplement: Supplementary Information [file srep44211-s1.pdf]

Visual detection and differentiation of Classic Swine Fever Virus strains using nucleic acid sequence-based amplification (NASBA) and G-quadruplex DNAzyme assay.

Xiaolu Lu[a], Xueyao Shi[b], Gege Wu[b], Tiantian Wu[b], Rui Qin[b], Yi Wang[b*]

[a] *School of Environmental Studies, China University of Geosciences (Wuhan), Wuhan 430074, P. R. China*

[b] *Ccollege of life sciences, South-central University for Nationalities, Wuhan 430074,P.R. China*

[*] Corresponding author

Tel: 86-27-67842689

E-mail: yiwang@mail.scuec.edu.cn

Supplementary Data

Figure S1. Hemin concentration optimization.

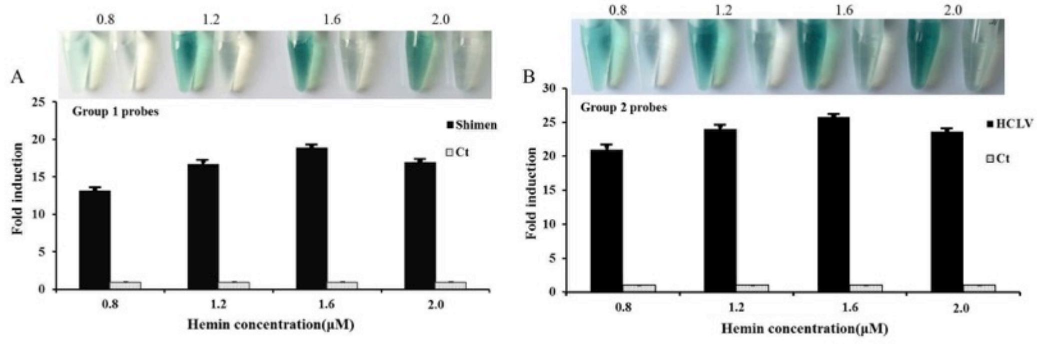

Different concentrations of hemin were tested in Shimen DNA (A) and HCLV DNA detection (B). Absorbance (OD) was measured at 414 nm and normalized to the value of negative control (Ct). The inset is the corresponding photograph of the color change. The data represent the mean ± S.D. of three independent experiments.

Figure S2. Probes concentration optimization.

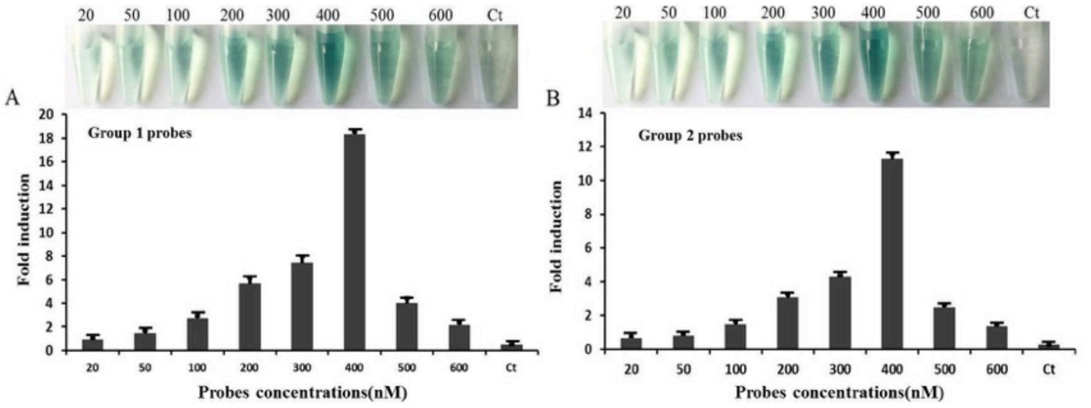

Different concentrations of probes A&B were tested in Shimen DNA (A) and HCLV DNA detection (B). Absorbance (OD) was measured at 414 nm and normalized to the value of negative control (Ct). The inset is the corresponding photograph of the color change. The data represent the mean ± S.D. of three independent experiments.

Figure S3. RNA template amount optimization.

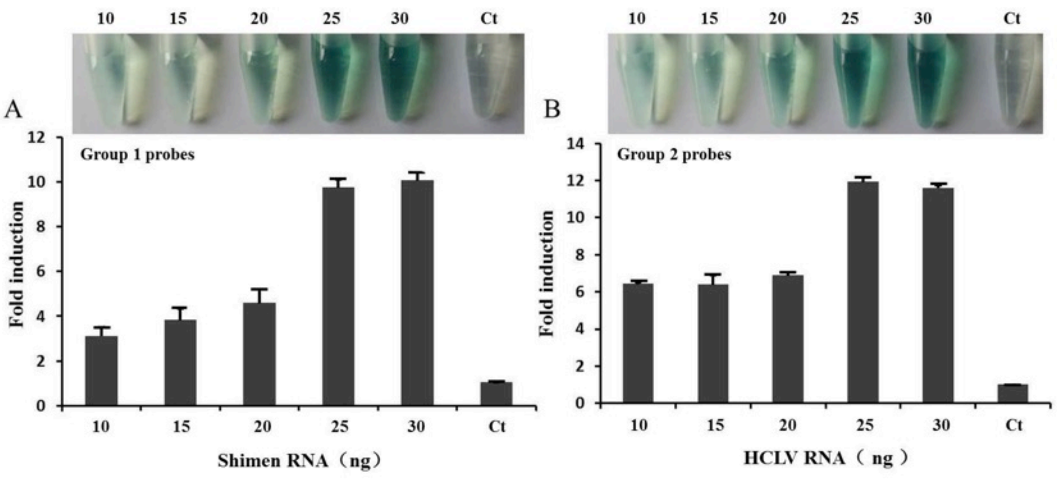

Different amount of purified RNA were tested in Shimen RNA (A) and HCLV RNA detection (B). Absorbance (OD) was measured at 414 nm and normalized to the value of negative control (Ct). The inset is the corresponding photograph of the color change. The data represent the mean ± S.D. of three independent experiments.

Figure S4. Probes optimization.

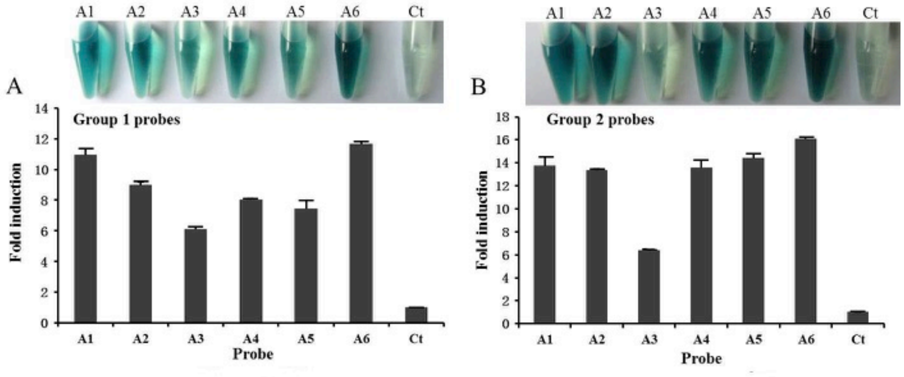

Different A probes were tested for Shimen DNA (A) and HCLV DNA detection (B). Absorbance (OD) was measured at 414 nm and normalized to the value of negative control (Ct). Ct: negative control with no template added to the detection system. The inset is the corresponding photograph of the color change. The data represent the mean ± S.D. of three independent experiments.

Figure S5. Sensitivity comparation between RT-PCR and NASBA in this study

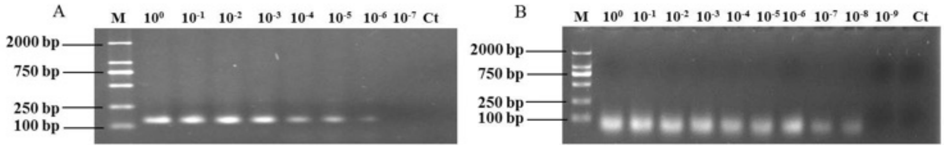

2 μg of total RNA extracted from CSFV-infected cells were serial diluted and subjected to either RT-PCR (A) or NASBA (B) to test the detect limit of these two method in this study.

Figure S6. Sequence alignment between CSFV wide-type strains and HCLV.

A

strain HCLV	gggtgagtatcagtactggtttgacctggatgcgactgaccgc
strain Shimen	gggcgagtatcagtactggtttgacctggacgtgacagaccgc
isolate Guizhou	gggcgagtatcagtactggtttgacctggacgtgacagaccgc
isolate CSFV-DaB-2008	gggcgagtatcagtactggtttgacctggacgtgacagaccgc
isolate CSFV-SS-2010	gggcgagtatcagtactggtttgacctggacgtgacagaccgc
isolate CSFV-PY-2009	gggcgagtatcagtactggtttgacctggacgtgacagaccgc
isolate CSFV-GZh-2009	gggcgagtatcagtactggtttgacctggacgtgacagaccgc
isolate CSFV-FS-2009	gggcgagtatcagtactggtttgacctggacgtgacagaccgc
isolate CSFV-DaB-2008	gggcgagtatcagtactggtttgacctggacgtgacagaccgc
isolate CSFV-XT-2010	gggcgagtatcagtactggtttgacctggacgtgacagaccgc

B

strain HCLV	gggtgagtatcagtactggtttgacctggatgcgactgaccgc
strain Shimen	gggcgagtatcagtactggtttgacctggacgtgacagaccgc
strain Koslov	gggcgagtatcagtactggtttgacctggacgtgactgaccgc
strain Thiverval	gggcgagtatcagtactggtttgacctggacgtgactgaccgc
strain Eystrup	gggcgagtatcagtactggtttgacctggacgtgactgaccgc
strain AlfortA19	gggcgagtatcagtactggtttgacctggacgtgactgaccgc
strain Alfort/187	gggcgagtatcagtactggtttgacctggacgtgactgaccgc
strain CAP	gggcgagtatcagtactggtttgacctggacgtgactgaccgc
strain ALD	gggcgagtatcagtactggtttgacctggacgtgactgaccgc
strain Glentorf	gggcgagtatcagtactggtttgacctggacgtgactgaccgc
strain LOM	gggcgagtatcagtactggtttgacctggacgtgactgaccgc

The alignment of the probe-detecting sequences of HCLV and 9 wild-type strains of CSFV in China (A), or of HCLV, Shimen and 9 wild-type strains of CSFV circulating outside China.
